# Paraneoplastic Neurological Syndromes: Advances and Future Perspectives in Immunopathogenesis and Management

**DOI:** 10.3390/antib15010008

**Published:** 2026-01-14

**Authors:** Stoimen Dimitrov, Mihael Tsalta-Mladenov, Plamena Kabakchieva, Tsvetoslav Georgiev, Silva Andonova

**Affiliations:** 1 Clinic of Rheumatology, University Multiprofile Hospital for Active Treatment “St. Marina”, 9010 Varna, Bulgaria; stoimen2000@gmail.com; 2 Second Clinic of Neurology with ICU and Stroke Unit, University Multiprofile Hospital for Active Treatment “St. Marina”, 9010 Varna, Bulgaria; mihael.tsalta@mu-varna.bg (M.T.-M.);; 3 Department of Neurology and Neuroscience, Faculty of Medicine, Medical University “Prof. Paraskev Stoyanov”—Varna, 9010 Varna, Bulgaria; 4 Clinic of Internal Diseases, Naval Hospital Varna, Military Medical Academy, 9010 Varna, Bulgaria; plamenakabakchieva@yahoo.com; 5 First Department of Internal Medicine, Faculty of Medicine, Medical University “Prof. Paraskev Stoyanov”—Varna, 9002 Varna, Bulgaria

**Keywords:** paraneoplastic neurological syndromes, autoimmune encephalitis, onconeural antibodies, immune checkpoint inhibitors

## Abstract

Paraneoplastic neurological syndromes (PNSs) are immune-mediated disorders caused by an antitumor response that cross-reacts with the nervous system, leading to severe and often irreversible neurological disability. Once considered exceedingly rare, PNSs are now increasingly recognized owing to the identification of novel neural autoantibodies, wider use of commercial testing, and the emergence of immune checkpoint inhibitor (ICI)-related neurotoxicity that phenotypically overlaps with classic PNS. In this narrative review, we performed a structured search of PubMed/MEDLINE, Scopus, Web of Science, and Google Scholar, without date restrictions, to summarize contemporary advances in the epidemiology, pathogenesis, diagnosis, and management of PNS. Population-based data show rising incidence, largely reflecting improved ascertainment and expanding indications for ICIs. Pathogenetically, we distinguish T-cell-mediated syndromes associated with intracellular antigens from antibody-mediated disorders targeting neuronal surface proteins, integrating emerging concepts of molecular mimicry, tumor genetics, and HLA-linked susceptibility. The 2021 PNS-Care criteria are also reviewed, which replace earlier “classical/non-classical” definitions with risk-stratified phenotypes and antibodies, and demonstrate superior diagnostic performance while underscoring that “probable” and “definite” PNS should be managed with equal urgency. Newly described antibodies and methodological innovations such as PhIP-Seq, neurofilament light chain, and liquid biopsy are highlighted, which refine tumor search strategies and longitudinal monitoring. Management principles emphasize early tumor control, prompt immunotherapy, and a growing repertoire of targeted agents, alongside specific considerations for ICI-associated neurological syndromes. Remaining challenges include diagnostic delays, limited high-level evidence, and the paucity of validated biomarkers of disease activity. Future work should prioritize prospective, biomarker-driven trials and multidisciplinary pathways to shorten time to diagnosis and improve long-term outcomes in patients with PNS.

## 1. Introduction

Paraneoplastic neurological syndromes (PNSs) represent a heterogeneous and clinically challenging group of immune-mediated disorders that affect the nervous system in patients with cancer [1]. Unlike complications arising from direct metastatic invasion, coagulopathy, metabolic derangements, or the neurotoxic effects of chemotherapy and radiation, these syndromes are remote effects of systemic malignancies driven by aberrant immune responses.

PNSs are characterized by the production of autoantibodies or cytotoxic T-cells directed against neural antigens that are ectopically expressed by tumor cells, a phenomenon that reflects a breakdown in immune tolerance [1]. While the immune system attempts to suppress tumor growth—a concept supported by the observation that patients with PNS often exhibit more indolent oncological progression—the cross-reactivity with healthy nervous tissue results in significant, often irreversible, neurological morbidity.

Historically, PNSs were considered exceedingly rare, with estimates suggesting they affected fewer than 1% of patients with cancer. However, the landscape of paraneoplastic disorders has undergone a profound transformation over the past two decades. The discovery of a rapidly expanding repertoire of neuronal autoantibodies, particularly those targeting cell-surface synaptic proteins, has redefined the epidemiology and phenotypic spectrum of these diseases [1,2].

Recent population-based data suggest a rising incidence, likely attributable to improved diagnostic vigilance and the widespread availability of commercial antibody panels rather than a true biological increase in paraneoplastic autoimmunity. Furthermore, the advent of immune checkpoint inhibitors (ICIs) in modern oncology has introduced a new category of neurological immune-related adverse events (n-irAEs), which phenotypically mimic spontaneous PNS but may possess distinct immunopathogenic underpinnings, creating a complex intersection between neuro-oncology and autoimmunity [3].

Despite these advances, significant diagnostic and management challenges persist. Epidemiological discrepancies between population data and observed incidence suggest ongoing under-recognition of these disorders. Moreover, the reliability of commercial diagnostic assays remains a critical concern, with recent studies highlighting the diagnostic pitfalls of commercial line blots and the necessity for confirmatory testing [4,5].

We aim to address these knowledge gaps by critically evaluating the latest diagnostic methodologies and therapeutic evidence, largely derived from observational cohorts due to the scarcity of randomized trials, to propose a cohesive framework for future clinical practice and research.

## 2. Search Strategy and Selection Criteria

In accordance with the principles of high-quality narrative reviews [6], we conducted a structured search to synthesize current evidence on PNS. We queried PubMed/MEDLINE, Scopus, and Web of Science for articles published in English, using a comprehensive Boolean strategy tailored to each database: (“paraneoplastic neurological syndromes” OR “autoimmune encephalitis” OR “onconeural antibodies” OR “paraneoplastic cerebellar degeneration”) AND (“diagnosis” OR “PNS-Care” OR “biomarkers” OR “PhIP-Seq”) AND (“immunotherapy” OR “immune checkpoint inhibitors” OR “management”). The search strategy prioritized original research, consensus guidelines, and systematic reviews that addressed recent advances in diagnostics and emerging therapeutics.

From an initial pool of 1942 records, 86 sources were selected for the final synthesis based on their relevance to the review’s thematic focus. Given the significant heterogeneity of the included literature-which consists predominantly of retrospective cohort studies, case series, and expert consensus rather than randomized controlled trials—a formal critical appraisal of evidence quality was not performed. Instead, data were extracted and synthesized narratively to provide a holistic overview of the field’s evolution and current clinical standards.

## 3. Etiology and Epidemiology

### 3.1. Incidence and Prevalence Trends

The epidemiology of PNS has historically been difficult to ascertain due to the rarity of these conditions and variability in diagnostic rigor. However, recent population-based studies have provided robust estimates of the disease burden (Table 1). Pivotal studies from Italy [2] and the United States [7] report a prevalence ranging between 4.4 and 5.4 per 100,000 inhabitants, with incidence rates consistently rising over the last decade. This upward trajectory—documented as doubling in some cohorts—strongly suggests improved ascertainment through wider autoantibody testing rather than a biological shift in risk. Furthermore, data from France indicate that the incidence of antibody-positive autoimmune encephalitis has recently surpassed that of classical PNS, reflecting the expanding recognition of non-paraneoplastic autoimmune etiologies in the era of modern biomarkers [8].

**Table 1:** Population-Based Epidemiology of PNS and Antibody-Mediated Encephalitis.

Study (Region)	Study Period	Incidence Rate	Prevalence	Key Findings
Vogrig et al. [2] (North-East Italy)	2009–2017	0.89 per 100,000 (Rising trend: 0.62 ⟶ 1.22)	4.37 per 100,000	Incidence doubled over 9 years, strongly suggesting improved ascertainment rather than biological risk shift.
Dubey et al. [7] (Olmsted County, USA)	1987–2018	0.6 per 100,000 (Rising trend: 0.4 ⟶ 0.8)	5.4 per 100,000	Validates the rising trend observed in Europe; confirms comparable disease burden across Western populations.
Hébert et al. [8] (France)	2016–2018	2.1 per million * (Antibody-positive AE only)	N/A	The incidence of antibody-mediated autoimmune encephalitis surpassed that of classical PNS.

Abbreviations: AE—autoimmune encephalitis; PNS—Paraneoplastic Neurological Syndrome; USA—United States of America. * Note: The French study reports incidence per million person-years, reflecting specific antibody-positive subtypes rather than the broader clinical definition used in other cohorts.

### 3.2. Demographic Variations

Demographic factors such as age and sex exert a profound influence on the clinical phenotype, antibody profile, and associated malignancy risk in PNS. Understanding these demographic nuances is essential for calculating pre-test probability and guiding the oncological search.

The median age of onset for most PNS is between 50 and 70 years, reflecting the peak age incidence of the associated malignancies such as lung cancer and breast cancer [2]. However, distinct bimodal distributions exist. Anti-N-methyl-D-aspartate receptor (NMDAR) encephalitis predominantly affects young women (median age ~25 years) when associated with ovarian teratomas, whereas late-onset cases (age > 45 years) are more gender-balanced and less likely to be paraneoplastic [9,10]. Conversely, syndromes associated with antibodies against the voltage-gated potassium channel (VGKC) complex proteins, such as Leucine-rich glioma-inactivated 1 (LGI1) and Contactin-associated protein-like 2 (CASPR2), are significantly more common in older males [11].

Female predominance is observed in syndromes associated with breast and gynecological malignancies, such as anti-Yo (PCA-1) cerebellar degeneration and anti-Ri (ANNA-2) opsoclonus-myoclonus [12,13]. In contrast, anti-Ma2 (Ta) encephalitis shows a strong male predilection due to its association with testicular germ cell tumors, particularly in men under 45 years of age [14]. The newly described anti-Kelch-like protein 11 (KLHL11) antibody also exhibits a strong male predominance, linking rhombencephalitis to testicular seminomas [15].

### 3.3. Oncological Associations

The distribution of underlying malignancies in PNS is non-random and is strongly predicted by the specific neuronal autoantibody present.

Small cell lung cancer (SCLC) is the most frequently associated tumor in PNS, accounting for a significant proportion of cases. Its neuroendocrine origin renders it highly immunogenic, as SCLC cells frequently express neuronal antigens such as HuD, CRMP5, and voltage-gated calcium channels (VGCC) [16]. SCLC is the primary driver of syndromes associated with anti-Hu (ANNA-1), anti-CV2/CRMP5, anti-amphiphysin, and anti-SOX1 antibodies [17,18,19,20]. Interestingly, anti-Hu seropositivity occurs in 16–25% of neurologically asymptomatic SCLC patients, albeit at much lower titers compared to those with clinical disease [21].

Thymic epithelial tumors are uniquely capable of generating autoreactive T-cells due to defective negative selection within the neoplastic thymus. Thymoma is the classic association for myasthenia gravis (AChR antibodies) but is also strongly linked to central nervous system (CNS) syndromes such as Morvan syndrome and limbic encephalitis (LE), mediated by anti-CASPR2, anti-LGI1, and anti-CRMP5 antibodies [19,22,23].

In women, breast cancer and ovarian malignancies are dominant triggers. Breast cancer is strongly linked to anti-Yo (PCA-1), anti-Ri (ANNA-2), and anti-amphiphysin antibodies [12,13,18]. Ovarian teratomas, which contain varying amounts of differentiated neuroglial tissue, are the quintessential trigger for anti-NMDAR encephalitis [24]. The presence of neural tissue within the teratoma, often infiltrated by immune cells organizing into tertiary lymphoid structures with germinal centers, provides a continuous source of antigen stimulation [25].

In young men, testicular germ cell tumors (TGCTs), particularly seminomas, are the leading cause of PNS. The classic association is with anti-Ma2 encephalitis, characterized by limbic, diencephalic, and brainstem dysfunction [26]. More recently, the discovery of anti-KLHL11 antibodies has resolved the etiology of many previously “seronegative” cases of brainstem encephalitis and ataxia in this population [15].

Hodgkin lymphoma is strongly associated with anti-Tr (DNER) antibodies, manifesting as paraneoplastic cerebellar degeneration (PCD), and anti-mGluR5 antibodies, causing “Ophelia syndrome” (LE). Non-Hodgkin lymphomas can be associated with paraneoplastic neuropathies and dermatomyositis [27].

Beyond these classic associations, isolated cases of PNS combined with diverse paraneoplastic clinical manifestations, involving other solid tumors have been reported. Ochi et al. described a rare presentation of gastric adenocarcinoma manifesting as posterior interosseous nerve palsy accompanied by symmetrical polyarthritis, both of which remitted after gastrectomy [28]. Similarly, Kobak reported an unusual case of prostate cancer presenting with paraneoplastic peroneal paresis and chronic monoarthritis, which resolved following tumor resection [29].

## 4. Pathogenesis

The pathogenesis of PNS is complex and involves a breach of immune tolerance to neural antigens. Current understanding categorizes pathogenesis into two primary mechanisms: cytotoxic T-cell-mediated damage (typically associated with intracellular antigens) and antibody-mediated dysfunction (associated with surface antigens).

### 4.1. Intracellular Antigens and T-Cell Cytotoxicity

For syndromes associated with “high-risk” antibodies targeting intracellular proteins, the antibodies themselves are generally considered non-pathogenic in vivo because they cannot access the cytoplasmic or nuclear compartments of live neurons [30]. Instead, these antibodies serve as highly specific biomarkers for a T-cell-mediated immune response. The primary pathogenic effectors are CD8+ cytotoxic T-cells that recognize neuronal peptides presented by Major Histocompatibility Complex (MHC) class I molecules on the surface of neurons [31].

Histopathology in anti-Hu and anti-Yo syndromes typically reveals extensive CD8+ T-cell infiltration, perivascular cuffing, microglial activation, and profound neuronal loss [30]. In anti-Yo PCD, for example, there is often a near-total loss of Purkinje cells with relative preservation of other cerebellar layers, accompanied by Bergmann gliosis and T-cell infiltrates. This irreversible neuronal destruction explains the generally poor neurological prognosis and limited response to immunotherapy observed in these syndromes [30].

The tumor microenvironment plays a critical role in initiating this response. Tumors from patients with PNS often show heavy infiltration by inflammatory cells, suggesting a robust anti-tumor immune response that cross-reacts with neural tissue [32]. This “onconeural” immunity may confer an oncological survival advantage; multiple studies have demonstrated that patients with PNS often have more limited metastatic spread and better tumor-specific survival compared to antibody-negative patients with the same cancer type [16].

### 4.2. Surface Antigens and Antibody-Mediated Dysfunction

In contrast, syndromes associated with antibodies against neuronal surface antigens (e.g., NMDAR, LGI1, GABA-B receptor, AMPAR) involve direct antibody-mediated pathogenicity [33]. These antibodies bind to extracellular domains of synaptic receptors, ion channels, or adhesion molecules, leading to internalization, cross-linking, or functional blockade of the target.

Histopathology in these cases is distinct from the T-cell pattern. Brain tissue often shows minimal neuronal death and less intense inflammation, with B-cell and plasma cell infiltration being more prominent than in intracellular syndromes [34]. In anti-NMDAR encephalitis, for instance, autopsy studies reveal a specific reduction in the density of NMDARs in the hippocampus without significant neuron loss, consistent with the antibody-mediated internalization mechanism. This preservation of neuronal integrity underpins the potential for significant recovery following immunotherapy and antibody depletion, even after prolonged disease courses [34].

A unique histological feature has been identified in ovarian teratomas associated with anti-NMDAR encephalitis. These tumors frequently contain neuroglial tissue infiltrated by immune cells that organize into tertiary lymphoid structures with functional germinal centers [24]. This finding suggests that the tumor itself acts as an ectopic site of antibody production and affinity maturation, contributing to the sustained autoimmune response and explaining why tumor resection is such a critical therapeutic intervention [1].

### 4.3. Immunogenetics and HLA Associations

Genetic susceptibility plays a significant role in determining which patients with cancer will develop PNS. Specific Human Leukocyte Antigen (HLA) alleles have been identified that likely facilitate the presentation of onconeural peptides to T-cells [35]. A strong association has been found with HLA-DQ2 and HLA-DR3 class II molecules in anti-Hu positive patients. These alleles are also linked to other autoimmune disorders like celiac disease [36]. In anti-Yo positive cases, a specific susceptibility haplotype involving HLA-DRB1*13:01 and HLA-DQA1*01:03 has been reported, particularly in patients with ovarian cancer, whereas the HLA-DRB1*04:01 allele appears to be protective [37]. Recent Genome-Wide Association Studies (GWAS) have confirmed an exceptionally strong association between anti-LGI1 antibodies and HLA-DRB1*07:01, which is thought to present the LGI1 peptide to helper T-cells with high affinity [38]. Anti-IgLON5 disease is strongly linked to the HLA-DRB1*10:01 and HLA-DQB1*05:01 alleles, serving as a bridge between autoimmunity and neurodegeneration [39]. Beyond HLA, GWAS are beginning to uncover non-HLA genetic risk factors. For example, variants in the PTPRD gene have been identified as novel risk loci for anti-LGI1 encephalitis, implicating presynaptic signaling pathways in disease susceptibility [38].

### 4.4. Molecular Mimicry and Tumor Genetics

**Figure 1:**
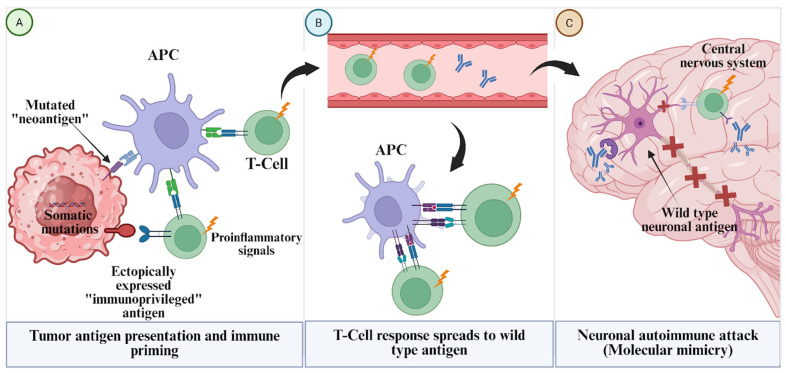
Schematic overview of the proposed PNS pathogenesis: Ectopic Antigen Expression and Tolerance Loss. (**A**) Current models suggest tumor cells ectopically express or overexpress neuronal antigens (e.g., CDR2L), which are presented to prime naïve T-cells. (**B**) Activated T-cells traffic to the CNS, potentially expanding their target range via epitope spreading. (**C**) This loss of tolerance is hypothesized to facilitate neuronal injury by cross-reactive T-cells and autoantibodies. Note: This diagram provides a simplified conceptual framework of these complex immunological cascades.

The concept of molecular mimicry and ectopic antigen expression is central to PNS pathogenesis (Figure 1). Typically, the immune system avoids attacking “self” antigens unless tolerance is compromised. In PNS, this compromise frequently arises because the tumor expresses a neuronal antigen within an immunogenic context or in an altered form [40]. Recent genomic studies have investigated whether tumors from patients with PNS harbor somatic mutations, amplifications, or structural alterations in the genes encoding the onconeural antigen. For example, while ovarian carcinomas in patients with anti-Yo PCD can harbor somatic mutations or amplifications in the CDR2L gene, these genetic alterations are present in a minority of cases [41]. Instead, the universal finding in these tumors is the intense overexpression of the onconeural protein (e.g., CDR2L) accompanied by a robust inflammatory microenvironment, which facilitates the breakdown of tolerance. These mechanisms may create “neoantigens” or present self-antigens in a pro-inflammatory context that are recognized as foreign by the immune system, triggering an initial T-cell response that subsequently spreads to the wild-type antigen expressed in the cerebellum. Similarly, the ectopic expression of “immunoprivileged” neuronal proteins (like Ma2) in the context of pro-inflammatory signals within the tumor microenvironment is a key trigger for the autoimmune cascade [32].

## 5. Clinical Characteristics

**Figure 2:**
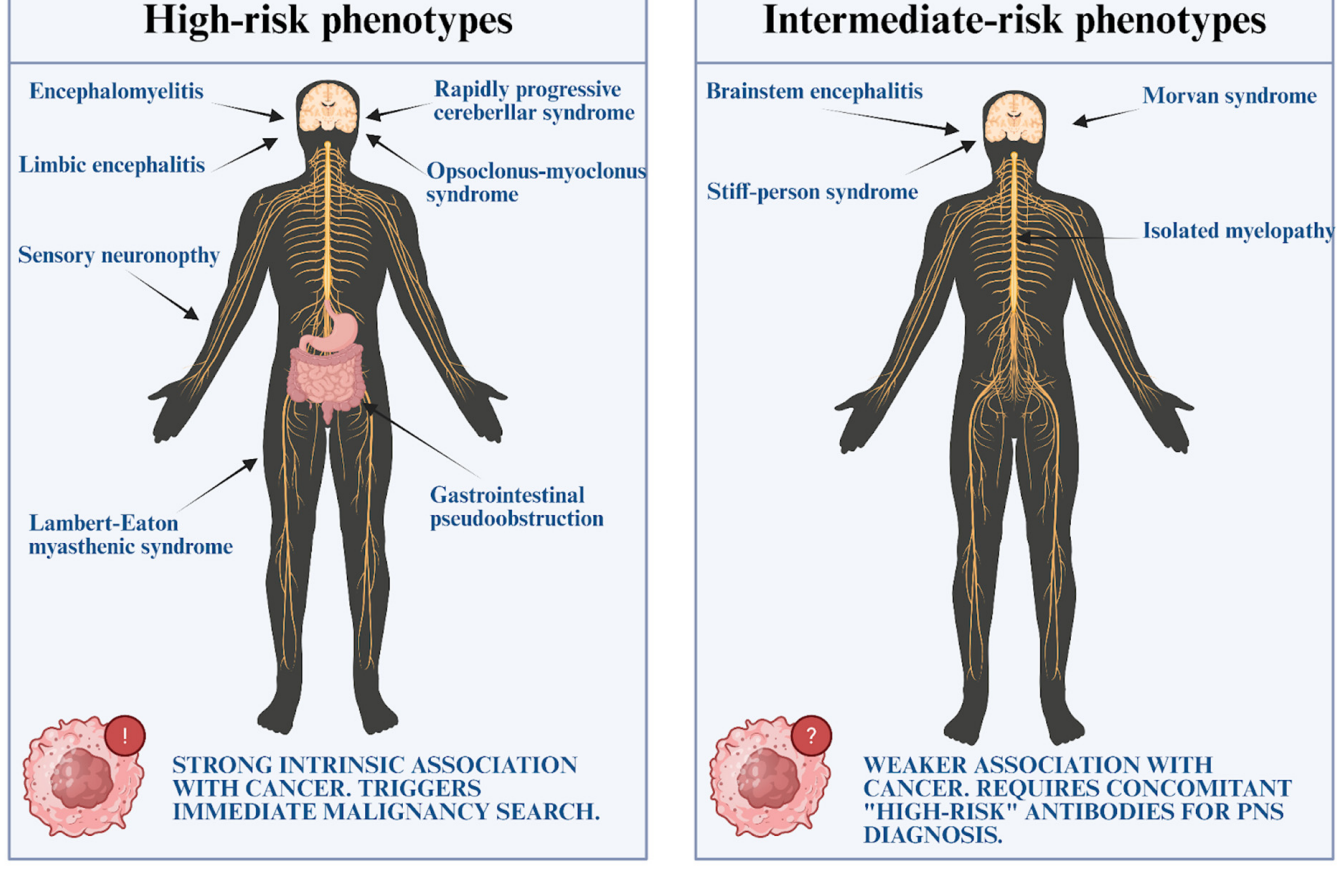
Stratification of Clinical Phenotypes according to the 2021 Updated Criteria. (**Left**) High-risk phenotypes exhibit a specific clinical pattern strongly associated with malignancy, warranting immediate screening. (**Right**) Intermediate-risk phenotypes show variable cancer associations and typically require the detection of high-risk antibodies (PNS-Care Score) to confirm a paraneoplastic etiology.

The clinical spectrum of PNS is vast, potentially affecting any level of the neuraxis from the cerebral cortex to the neuromuscular junction. To standardize diagnosis, the updated 2021 criteria stratify clinical presentations into two distinct categories: “High-risk phenotypes” and “Intermediate-risk phenotypes” [42] (Figure 2). The former represents the classical syndromes with a strong, intrinsic association with cancer, whereas the latter encompasses conditions that, while potentially paraneoplastic, are frequently idiopathic or autoimmune in origin without underlying malignancy.

### 5.1. High-Risk Phenotypes

Recognition of high-risk phenotypes mandates immediate malignancy screening, often independent of antibody confirmation. Encephalomyelitis, the archetypal syndrome associated with anti-Hu antibodies and SCLC, involves multifocal inflammation spanning the brain to the dorsal root ganglia, presenting with cognitive decline, seizures, and sensory deficits [17]. When restricted to the medial temporal lobes, Limbic Encephalitis (LE) manifests with subacute memory deficits, temporal seizures, and psychiatric symptoms [43]. Rapidly progressive cerebellar syndrome is characterized by severe pancerebellar dysfunction (ataxia, dysarthria, nystagmus) that can render patients bedbound within weeks [12]. It is strongly linked to anti-Yo (ovarian/breast cancer) and anti-Tr/DNER (Hodgkin lymphoma) antibodies [13]. Opsoclonus-myoclonus syndrome (OMS) involves chaotic saccades and myoclonus; in adults, it is associated with breast cancer (anti-Ri) and SCLC, whereas in children, it is the most common PNS, typically linked to neuroblastoma [44,45].

Peripheral manifestations include Sensory Neuronopathy (SNN), a hallmark of anti-Hu syndrome causing asymmetric, non-length-dependent sensory loss due to dorsal root ganglion destruction [17,46]. In the autonomic domain, gastrointestinal pseudo-obstruction results from autoimmune attacks on the myenteric plexus, leading to obstruction without mechanical cause [47]. Finally, Lambert-Eaton myasthenic syndrome (LEMS) is a presynaptic disorder of the neuromuscular junction causing proximal weakness that improves with exercise. Approximately 60% of cases are paraneoplastic, almost exclusively associated with SCLC [20,48].

### 5.2. Intermediate-Risk Phenotypes

Unlike classical syndromes, intermediate-risk phenotypes are not intrinsically indicative of cancer and often have idiopathic counterparts. Consequently, diagnosing them as PNS requires the detection of high-risk antibodies [42]. Brainstem encephalitis manifests with cranial nerve deficits and central hypoventilation. When linked to anti-Ma2 antibodies (testicular cancer), inflammation often extends to the diencephalon, causing distinctive narcolepsy and hyperphagia [14]. Morvan Syndrome bridges the CNS and PNS, characterized by peripheral nerve hyperexcitability (neuromyotonia) alongside severe insomnia (agrypnia excitata), dysautonomia, and encephalopathy. It is strongly linked to anti-CASPR2 antibodies and thymoma [11,22]. Finally, Stiff-Person Syndrome (SPS) is typically non-paraneoplastic (anti-GAD65), but paraneoplastic variants occur, notably in women with breast cancer and anti-amphiphysin antibodies [49]. Isolated myelopathy, often presenting as longitudinally extensive transverse myelitis (LETM), warrants malignancy screening when associated with anti-CRMP5 or anti-amphiphysin antibodies [50].

### 5.3. ICI-Associated Neurological Syndromes

The widespread use of ICIs has introduced a new spectrum of n-irAEs affecting 1–12% of patients [3]. Clinically, these disorders often present with unique high-risk phenotypes. A critical example is the “Triple M” syndrome (myositis, myocarditis, myasthenia gravis), which carries a mortality rate exceeding 50% due to cardiorespiratory failure and demands immediate recognition [51]. Other distinct manifestations include aseptic meningitis and ICI-associated vestibulopathy [52]. Regarding immunopathogenesis, n-irAEs diverge from classic PNS: while the latter relies on specific onconeural antibodies, n-irAEs frequently arise from generalized, non-specific T-cell disinhibition. However, these mechanisms intersect when ICIs “unmask” latent autoimmunity in patients with pre-existing, low-titer antibodies, precipitating fulminant PNS [53]. This risk highlights the potential utility of screening for high-risk antibodies before starting ICI therapy [42].

## 6. Advances in Diagnostics and Novel Antibodies

The diagnostic framework for PNS was fundamentally revised in 2021 by the PNS-Care Panel, a group of international experts who updated the 2004 consensus criteria [42,54]. This update marked a shift away from the binary classification of “classical” versus “non-classical” syndromes and “onconeural” versus “non-onconeural” antibodies. Instead, the new criteria utilize a risk-stratification model based on “High-Risk” and “Intermediate-Risk” phenotypes and antibodies, culminating in the PNS-Care Score. This scoring system integrates clinical presentation, antibody specificity, and oncological findings to provide a level of diagnostic certainty—“Definite, Probable, or Possible PNS”—thereby standardizing clinical decision-making and research inclusion criteria.

### 6.1. The 2021 PNS-Care Score

The 2021 PNS-Care criteria shifted PNS diagnosis from a binary “classical/non-classical” classification to a probabilistic, data-driven framework. This scoring system was developed to standardize clinical decision-making and research inclusion, addressing the critical need for rigorous case definitions in an era of expanding antibody discovery and frequent off-label use of commercial diagnostic panels. The criteria integrate three key domains—clinical phenotype, antibody risk level, and oncological evidence—into a cumulative score that stratifies patients into “Possible” (4–5 points), “Probable” (6–7 points), or “Definite” (≥8 points) PNS categories [42]. A pivotal change in this system is the replacement of the term “onconeural” with risk-stratified antibody categories, distinguishing “High-risk” antibodies (>70% cancer association, e.g., anti-Hu, anti-Yo) from “Intermediate-risk” antibodies (30–70% cancer association, e.g., anti-NMDAR, anti-GABA-B receptor), thereby reflecting the nuanced oncological associations of surface-directed antibodies [42].

Recent large-scale retrospective validation has confirmed the utility of this updated framework. In a cohort of 484 patients, the 2021 criteria (using a cutoff of Definite or Probable, Score ≥ 6) achieved a sensitivity of 93% and specificity of 100%, significantly outperforming the 67% sensitivity of the 2004 criteria [55]. This enhancement is largely attributable to the incorporation of newly identified biomarkers absent from previous guidelines. However, the study also revealed important limitations: 15 false-negative cases (Score ≥ 5) were identified, primarily involving anti-KLHL11 brainstem encephalitis. These cases often failed to reach the “Probable” threshold due to the antibody’s “Intermediate-risk” classification and the difficulty of detecting the associated “burned-out” testicular tumors, highlighting a specific blind spot in the current scoring system [55].

### 6.2. Novel and Emerging Antibodies

The application of advanced discovery techniques has led to the identification of several new high-risk antibodies, resolving the etiology for many patients previously labeled as “seronegative.” Anti-KLHL11 was identified in 2019 using Phage ImmunoPrecipitation Sequencing (PhIP-Seq). This antibody targets Kelch-like protein 11, an intracellular protein involved in ubiquitination. It is strongly associated with testicular seminoma and presents as a rhombencephalitis (brainstem/cerebellar symptoms) with hearing loss and ataxia, primarily in men [15]. It represents a major diagnostic breakthrough for young men with testicular cancer and neurological symptoms [56,57]. Antibodies against Seizure-related 6 homolog like 2 (Anti-Sez6l2) have been linked to cerebellar ataxia and retinopathies, associated with various carcinomas including breast and lung [58]. Anti-IgLON5 disease was initially described as a primary neurodegenerative tauopathy with sleep dysfunction. Presently it is recognized to have a paraneoplastic etiology in a subset of patients, associated with lung and breast cancers [59]. Anti-SKOR2 is another recently identified antibody using PhIP-Seq, linked to paraneoplastic cerebellar degeneration and adenocarcinomas [60].

### 6.3. Methodological Advances: PhIP-Seq and Biomarkers

**Figure 3:**
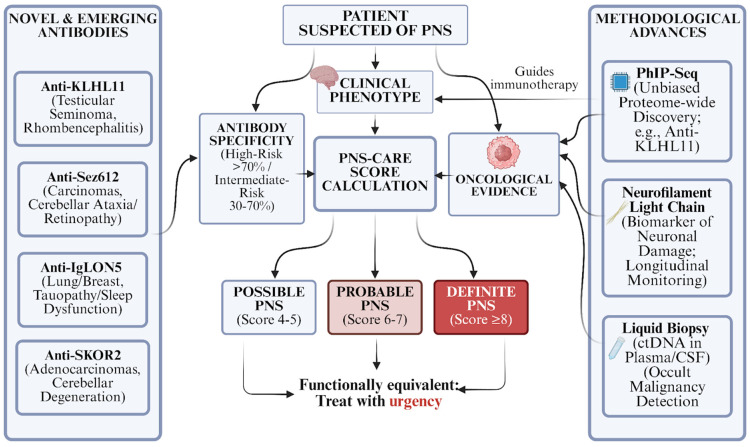
Integrated Diagnostic Workflow for PNS. The updated PNS-CARE framework combines clinical phenotyping, antibody risk stratification, and oncological screening to classify patients (Possible, Probable, Definite). While traditional screening (IHC/Line Blot) remains standard, the integration of PhIP-Seq for novel antibody discovery, Serum NfL for prognostication, and Liquid Biopsy (CSF/Plasma) for occult tumor detection significantly enhances diagnostic precision and monitoring capabilities.

The traditional diagnostic workflow relies on tissue immunohistochemistry (IHC) screening followed by confirmatory immunoblot or cell-based assays (CBA) [42]. However, commercial line blots are prone to false positives, particularly for bands like Yo and Ma2, necessitating caution in interpretation [4,5] (Figure 3).

The PhIP-Seq technique allows unbiased, proteome-wide autoantibody discovery [61]. It utilizes bacteriophage libraries that display hundreds of thousands of human peptide sequences. Patient serum is incubated with the library, and antibodies bind to their specific peptide targets on the phages. The bound phages are then immunoprecipitated and sequenced to identify the enriched antigens. PhIP-Seq was instrumental in the discovery of anti-KLHL11 [57] and continues to uncover novel targets in seronegative PNS cohorts [62]. Serum NfL levels have emerged as a sensitive, nonspecific biomarker of neuroaxonal injury validated across various neurological conditions [63]. In the context of cancer patients with acute neurological complications, including paraneoplastic syndromes and ICI-neurotoxicity, elevated baseline levels have been associated with disease severity and higher mortality, while longitudinal monitoring may assist in gauging clinical recovery and response to treatment [64]. Furthermore, recent data in anti-IgLON5 disease demonstrate that serum NfL correlates with clinical severity scores and independently predicts 1-year mortality, highlighting its emerging prognostic value in specific PNS subtypes [65]. The analysis of circulating tumor DNA (ctDNA) in plasma or cerebrospinal fluid (CSF) (“liquid biopsy”) offers a new avenue for detecting occult malignancies in patients with PNS [66,67]. This technology could be particularly valuable for patients with high-risk antibodies but negative conventional imaging, potentially identifying microscopic tumors earlier in the disease course.

## 7. Management

PNS management is dual-pronged: rapid tumor treatment to remove the antigen source, and aggressive immunotherapy to prevent neurological damage.

### 7.1. Oncological Management

Tumor removal is the single most effective treatment for PNS and is associated with the best long-term neurological outcomes. In anti-NMDAR encephalitis, early resection of the ovarian teratoma is a robust predictor of recovery and reduced relapse risk [68]. For malignant tumors like SCLC, prompt initiation of chemotherapy and radiation is critical. In cases where a high-risk antibody is present but no tumor is found on imaging, aggressive surveillance is mandated. Some experts discuss exploratory laparoscopy in selected high-risk anti-Yo cases when imaging remains negative, though this is not universally recommended [42].

### 7.2. Immunotherapy

Immunotherapeutic strategies are stratified based on the severity of the syndrome and the underlying pathogenic mechanism (antibody-mediated vs. T-cell mediated).

First-line Therapy typically involves acute induction with high-dose intravenous corticosteroids (methylprednisolone), Intravenous Immunoglobulin (IVIG), and/or Plasma Exchange (PLEX) [69]. These modalities aim to rapidly lower antibody levels and suppress the underlying inflammatory process. If first-line therapy is unsuccessful, options include Rituximab (an anti-CD20 monoclonal antibody) and Cyclophosphamide. Rituximab is particularly effective for syndromes driven by B-cells and surface antibodies (e.g., NMDAR, LGI1) [70]. Cyclophosphamide is typically considered for T-cell mediated intracellular syndromes or refractory disease, given its ability to target broad lymphocyte proliferation, although efficacy in these conditions remains limited [71].

### 7.3. Emerging and Experimental Therapies for Refractory Disease

For patients with refractory disease, several novel agents targeting specific immune pathways are showing promise (Table 2).

Bortezomib is a proteasome inhibitor widely used in multiple myeloma. It depletes long-lived plasma cells, which are the primary source of autoantibodies and are CD20-negative (thus resistant to rituximab). It has shown efficacy in refractory anti-NMDAR encephalitis [72] and has been described in single reports of severe stiff-person syndrome, leading to clinical improvement and reduction in antibody titers [73]. By antagonizing the IL-6 receptor, tocilizumab blocks the inflammatory cascade and B-cell survival signals. It has demonstrated efficacy in autoimmune encephalitis refractory to rituximab, serving as a critical alternative for patients who fail to respond to standard B-cell depleting therapies [74]. FcRn Inhibitors like Efgartigimod block the neonatal Fc receptor, preventing the recycling of IgG and leading to a rapid reduction in circulating antibody levels. They are approved for myasthenia gravis based on pivotal Phase 3 data [75]. Their potential utility in other IgG4-mediated PNS, such as anti-CASPR2, anti-LGI1 and anti-IgLON5 disease, is currently suggested by their shared immunopathogenesis and described in isolated case reports [76]. Daratumumab is an anti-CD38 monoclonal antibody that targets plasma cells. It has been used successfully in severe refractory cases of NMDAR encephalitis and other antibody-mediated syndromes where bortezomib has failed or is contraindicated [77,78]. Janus Kinase (JAK) inhibitors (e.g., tofacitinib, ruxolitinib) block downstream cytokine signaling pathways. While evidence in classic PNS is limited, they have demonstrated efficacy in severe ICI-associated neurotoxicity [79] and are being explored for refractory inflammatory epilepsy syndromes like NORSE [80]. Their application in PNS remains experimental, relying on the rationale of broad cytokine suppression in T-cell mediated autoimmunity. CD19-targeted Chimeric Antigen Receptor T-cell (CAR-T) therapy involves the genetic engineering of autologous T-cells to recognize and eliminate B-cells. While currently established only in hematological malignancies, it is being explored experimentally for severe, treatment-refractory autoimmune neurologic diseases [81]. Recent evidence highlights its potential utility in refractory Stiff-Person Syndrome, where it induced deep B-cell depletion and substantial clinical improvement in a treatment-resistant case, suggesting a future role for high-burden antibody-mediated phenotypes [82].

### 7.4. Management of ICI-Induced PNS

The management of n-irAEs requires a distinct approach guided by ASCO and other oncology society guidelines. For Grade 1 toxicities, ICI therapy may continue with close monitoring. For Grade 2, withholding the ICI and initiating corticosteroids is recommended. For Grade 3–4 toxicities (severe weakness, encephalitis), permanent discontinuation of the ICI is usually required, along with aggressive immunosuppression (IVIG, PLEX, or stronger agents like infliximab or tocilizumab) [83]. Re-challenging with ICIs after a neurological adverse event is high-risk, with significant relapse rates, particularly in patients with pre-existing paraneoplastic antibodies [53].

**Table 2:** Emerging Therapies for Refractory PNS and ICI-mediated neurotoxicity.

Therapy Class	Agent	Mechanism of Action	Potential Utility
Proteasome Inhibitor	Bortezomib	Depletes plasma cells (antibody factories)	Refractory anti-NMDAR, SPS; antibody-mediated PNS [72,73].
IL-6 Inhibitor	Tocilizumab	Blocks IL-6 receptor; reduces B-cell survival/inflammatory cascade	Refractory AE, ICI-induced cytokine release syndromes [74].
FcRn Inhibitor	Efgartigimod	Blocks neonatal Fc receptor; increases IgG catabolism	IgG4-mediated syndromes (e.g., LGI1, IgLON5), Myasthenia Gravis [75,76].
Anti-CD38 mAb	Daratumumab	Depletes plasma cells	Severe refractory antibody-mediated PNS [77,78].
JAK Inhibitor	Tofacitinib, Ruxolitinib	Inhibits Janus Kinase pathways	Potential for T-cell mediated inflammation (experimental) [79,80]).
CAR-T Therapy	Anti-CD19 CAR-T	Deep depletion of B-cell lineage	Experimental for severe refractory antibody-mediated PNS [82].

Abbreviations: AE, autoimmune encephalitis; anti-NMDAR, anti–N-methyl-D-aspartate receptor; CAR-T, chimeric antigen receptor T-cell; FcRn, neonatal Fc receptor; IgG, immunoglobulin G; IgG4, immunoglobulin G subclass 4; ICI, immune checkpoint inhibitor; IL-6, interleukin-6; JAK, Janus kinase; LGI1, leucine-rich glioma-inactivated protein 1; mAb, monoclonal antibody; PNS, paraneoplastic neurological syndromes; SPS, stiff-person syndrome.

## 8. Future Perspectives and Knowledge Gaps

Despite significant progress, the field of PNS faces critical challenges that require a prioritized research agenda. The most pressing unmet need is the reduction in diagnostic latency. Patients often face delays spanning months to years—averaging 5.4 years for sensory neuronopathy phenotypes—a window during which irreversible neuronal loss occurs [84]. Bridging this gap requires not only wider access to comprehensive antibody testing but also the validation of earlier biomarkers, such as liquid biopsy, to trigger screening before irreversible neurological damage occurs.

The second priority is the transformation of clinical trial design. High-quality randomized controlled trials (RCTs) have historically been hindered by the rarity of individual PNS subtypes. To overcome this, future research must shift from single-disease models to “basket trials” that group patients by shared immunopathogenesis (e.g., surface-antibody vs. intracellular T-cell mediated) rather than specific antibody targets [81]. This mechanistic grouping is the most tractable path to testing emerging agents like FcRn inhibitors or complement blockers with adequate statistical power.

Finally, a critical knowledge gap remains regarding the long-term burden of disease. Detailed health economic data and quality-of-life metrics for PNS survivors are sparse. As mortality rates improve with better acute therapies, research must pivot to address the chronic sequelae—cognitive impairment, ataxia, and pain—that continue to impose a heavy toll on patients and healthcare systems [85].

## 9. Strengths and Limitations

This review possesses several strengths that reflect the complexity of modern neuro-oncology. First, it offers a holistic examination of the entire clinical pathway, specifically highlighting the persistent challenges in early diagnosis and long-term monitoring. Rather than focusing solely on established diagnostic algorithms, the text critically evaluates the “grey zones” of clinical practice—such as distinguishing paraneoplastic mechanisms from immune checkpoint inhibitor toxicity—providing practical insights for complex case management. Second, the review integrates recent advances in pathophysiology, specifically the distinction between cytotoxic T-cell and antibody-mediated mechanisms, to explain the variance in treatment responsiveness. This mechanistic approach allows for a more nuanced discussion of therapeutic limitations and the realistic expectations for novel interventions.

However, several limitations must be acknowledged. As a narrative review, the selection of literature is subject to inherent bias and may not capture the exhaustive scope of a systematic meta-analysis. Crucially, while we endorse the 2021 PNS-Care criteria, their real-world implementation is hampered by limited access to advanced testing (e.g., PhIP-Seq, cell-based assays) and the risk of false positives in commercial line blots [4], which can lead to misdiagnosis in non-specialized settings. Furthermore, although we emphasize early diagnosis, it is important to recognize that for syndromes driven by intracellular antigens, prompt recognition does not always translate to clinical recovery. In these phenotypes, neurological deficits frequently stem from irreversible cytotoxic neuronal destruction that persists despite aggressive immunotherapy. Finally, regarding emerging interventions such as CAR-T cells and FcRn inhibitors, we must caution that current data are largely preliminary, derived primarily from case series rather than randomized controlled trials. Consequently, the broader application of these novel agents is currently constrained by undefined safety profiles and economic barriers, necessitating a careful risk-benefit assessment when treating older patients with active malignancies.

## 10. Conclusions

The characterization of PNS has advanced significantly, evolving into a distinct discipline at the convergence of neuro-oncology and immunology. While the identification of novel autoantibodies and the implementation of the 2021 PNS-Care criteria have standardized diagnostic rigor, the field currently faces a critical disconnect between scientific discovery and clinical outcomes. For syndromes associated with intracellular antigens, the frequent irreversibility of neuronal damage underscores that prognostic improvement relies less on novel therapeutics and more on the development of earlier screening strategies to facilitate intervention before overt neurodegeneration occurs.

Therapeutically, the future lies in shifting from broad empiric immunosuppression toward mechanism-based precision. The distinction between antibody-mediated and cytotoxic T-cell pathogenesis is fundamental to guiding treatment selection. To address the scarcity of high-level evidence, future research priorities should shift toward “basket trial” designs that group patients by immunophenotype rather than specific antibody targets, offering a feasible pathway to validate novel interventions in rare populations. Finally, as immune checkpoint inhibitors reshape oncological care, the rigorous differentiation between classic paraneoplastic mechanisms and iatrogenic neurotoxicity will define the next era of management. Ultimately, improving survivorship in this complex patient population requires a holistic approach that prioritizes neuro-preservation alongside oncological control.

## Data Availability

No new data were created in this study.
